# Transcriptional Profiling at High Temporal Resolution Reveals Robust Immune/Inflammatory Responses during Rat Sciatic Nerve Recovery

**DOI:** 10.1155/2017/3827841

**Published:** 2017-04-12

**Authors:** Lingyan Xing, Qiong Cheng, Guangbin Zha, Sheng Yi

**Affiliations:** Key Laboratory of Neuroregeneration of Jiangsu and Ministry of Education, Co-Innovation Center of Neuroregeneration, Nantong University, Nantong 226001, China

## Abstract

After peripheral nerve injury, immune/inflammatory responses are triggered, which are critical for nerve regeneration. Despite their importance, the underlying molecular changes in immune/inflammatory responses remain largely unknown. In this study, we systematically analyzed differentially expressed genes in immune/inflammatory-related pathways at high temporal resolution and experimentally validated gene expression changes with RT-PCR following sciatic nerve crush in rats. We found that immune/inflammatory reactions not only occur in the acute injury but also remained activated over two weeks after injury. Detailed bioinformatic studies suggested that multiple immune/inflammatory pathways, including agranulocyte adhesion and diapedesis, granulocyte adhesion and diapedesis, IL-6 signaling, and IL-10 signaling, were sustained activated during nerve degeneration and regeneration. Our current study expands our understanding of the molecular basis of altered immune/inflammatory-related pathways following injury and thus might offer the possibility of targeting related molecules as therapeutic intervention for peripheral nerve regeneration.

## 1. Introduction

Compared to the axons in the central nervous system, the peripheral nerves in adult mammals have great capacity for self-repair and regeneration. Successful peripheral regeneration depends on both the intrinsic growth capacity of peripheral axons and permissive microenvironment for axon growth [1, 2].

Peripheral nerve regeneration is a complex process that includes molecular changes in the immune/inflammatory pathways. In the process of axon degeneration, known as Wallerian degeneration, activated Schwann cells release cytokines and chemokines, which recruit the macrophages at the injury sites [3]. Accumulated macrophages further remove axonal and myelin debris, clearing a path for the subsequent axon regeneration [4]. Following peripheral nerve injury, both the innate and the acquired immune system are activated [5, 6]. Taken together, these previous studies indicated the importance of immune/inflammation responses in nerve injury.

Molecular changes in immune/inflammatory pathways following peripheral nerve injury were also identified. For example, the expression of various cytokines, including tumor necrosis factor (TNF), interleukin-6 (IL-6), and IL-10, are altered after nerve injury [7–11]. However, systematic analysis of gene expression changes in immune/inflammatory pathways and the related biological pathways during peripheral nerve injury and regeneration remain largely unclarified. In most cases, the functional recovery of severe peripheral nerve injury remains far from satisfactory. Therefore, it is necessary to obtain a better understanding of the roles of immune and inflammatory responses, which might help us find the molecular targets for posttraumatic nerve regeneration.

Our focus in this manuscript was on analyzing the molecular events in immune/inflammatory pathways following peripheral nerve injury in detail. Our previous work found that inflammatory and immune response-related functions, for example, immune cell trafficking and inflammatory response, were the top enriched categories following nerve injury [12]. Considering the importance of immune/inflammatory responses, in the current study, we further studied canonical pathways in immune/inflammation in detail. We found that four immune/inflammatory-related pathways, including agranulocyte adhesion and diapedesis, granulocyte adhesion and diapedesis, IL-6 signaling, and IL-10 signaling, were sustained activated during the whole process of nerve regeneration. mRNA expression of multiple molecules in these pathways were further validated by RT-PCR.

## 2. Materials and Methods

### 2.1. Ethics Statement, Animal Surgery, and RNA Preparation

All animal procedures were performed in accordance with the guidelines of Nantong University and ethically approved by the Administration Committee of Experimental Animals, Jiangsu Province, China.

Animal surgery was performed as previously described [12]. Briefly, adult male Sprague Dawley (SD) rats, weighting 180–220 g, were randomly divided into 5 groups: 0, 1, 4, 7, and 14 days post injury. 75 rats were used for deep sequencing [12], while another 30 rats were used for RT-PCR. Anaesthetized animals were cut a skin incision in the left outer mid-thigh. The sciatic nerve was crushed three times for 10 seconds each by using a sterilized forceps. The muscle and skin were then sutured. The rats in the sham group underwent sham surgery on the left sciatic nerve prior to nerve samples being harvested. Sciatic nerve segments of 5 mm in length at the crush site were harvested and stored at −80°C. Total RNA was extracted from harvested nerve as previously described [12].

### 2.2. RNA Sequencing and Analysis

The deep sequencing performed was described in the previous paper [12]. Following deep sequencing, expression levels of mapped genes were calculated and normalized by reads per kilobase transcriptome per million mapped reads (*RPKM*) to eliminate the influence of gene lengths and sequencing discrepancy. Briefly, *RPKM* value of single gene was calculated with this formula: *RPKM* = (10^9^ × *C*)/(*N* × *L*). *C* is the number of reads uniquely aligned to a particular gene, *N* is the total number of reads uniquely aligned to all genes, and *L* is the number of bases on a particular gene. Genes with a false discover rate (FDR) ≤ 0.001 and a fold change > 2  or < −2 are considered as differentially expressed at 1, 4, 7, and 14 days post sciatic nerve crush compared to sham group.

### 2.3. Bioinformatic Analysis

Differentially expressed genes were further uploaded to QIAGEN's Ingenuity® Pathway Analysis (IPA®, QIAGEN) and analyzed according to the Ingenuity Pathways Knowledge Base (IPKB). Differentially expressed genes (DEGs) were subjected to diseases and cellular functions and canonical signaling pathway analysis. DEGs in agranulocyte adhesion and diapedesis, granulocyte adhesion and diapedesis, IL-10 signaling, and IL-6 signaling were further analyzed and illustrated by the Venn diagram. A heatmap was generated using top over-represented DEGs based on the significance values (*p* values).

### 2.4. Real-Time PCR

RNA samples from 30 rats (different samples from what have been described in transcriptome sequencing) were used for real-time PCR to validate the results of transcriptome sequencing. The individual RNA sample (0.5 *μ*g) was reverse transcribed to cDNA with the Prime-Script reagent Kit (TaKaRa, Dalian, China) and further amplified with SYBR Green Premix Ex Taq (TaKaRa) in Applied Biosystems Stepone machine with listed primer pairs (see Additional File 1 available online at https://doi.org/10.1155/2017/3827841). Relative expression levels of mRNA were further quantified via the comparative 2^−ΔΔCt^ method, in which 18S rRNA was used as a reference gene. For more details, check [12].

### 2.5. Statistical Analysis

Experimental outcomes were represented as mean ± SEM and all data were analyzed using GraphPad Prism 6.0 (GraphPad Software Inc.). One-way ANOVA was used to compare multiple groups and a *p*  value < 0.05 was considered as significantly different.

## 3. Results

### 3.1. Diseases and Cellular Functions Analysis after Nerve Injury

Previously, we used a rat sciatic nerve crush model to analyze transcript changes from deep sequencing at 1, 4, 7, and 14 days following injury. We found 13,721, 14,321, 14,745, and 6979 DEGs compared to sham group, respectively [12].

**Figure 1:**
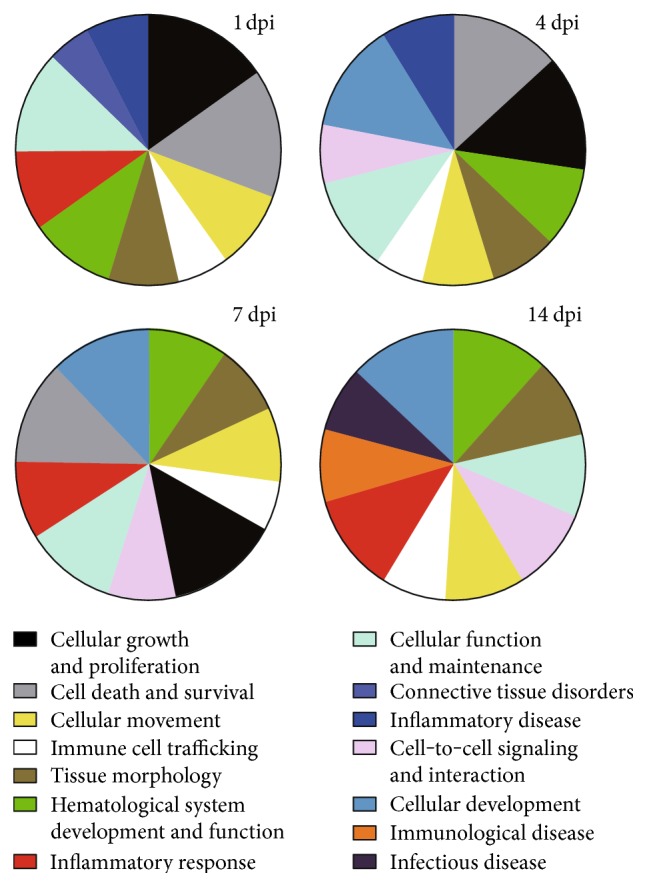
Diseases and cellular functions of differentially expressed genes (DEGs) following nerve injury by IPA analysis. Top 10 diseases and biofunctions to be overrepresented by DEGs at 1, 4, 7, and 14 days post injury (dpi). The size of sectors is correlated to the number of DEGs.

We applied IPA to analyzed diseases and functions with DEGs. Top of these DEGs at injured nerves were highly enriched in hematological system development, cellular movement, inflammatory response, and immune cell trafficking (Figure 1, Additional Files 2, 3, and 4, and [12]). Most diseases and function were activated following nerve injury (Additional Files 2, 3, and 4). We noticed that DEGs were enriched in immune/inflammatory responses at all four time points (1, 4, 7, and 14 days after injury) (Figure 1) [12].

### 3.2. Immune/Inflammatory-Related Canonical Signaling Pathways Post Nerve Injury

Further IPA canonical pathway analysis reveals that significantly dysregulated pathways in immune/inflammatory reactions following injury were agranulocyte adhesion and diapedesis, granulocyte adhesion and diapedesis, IL-10 signaling, and IL-6 signaling (Table 1, Additional Files 5, 6, 7, 8, 9, 10, 11, and 12) based on the score (−log (*p* value)) of each pathway (*p*  value < 0.05 were considered statistically significant). Agranulocyte/granulocyte adhesion and diapedesis are primary lines of host defense against infection, critical for recruiting agranulocytes and granulocytes to the injury sites. IL-6 is a proinflammatory cytokine, while IL-10 is an anti-inflammatory cytokine which limit and eliminate proinflammatory activity. Interestingly, pathways of agranulocyte adhesion and diapedesis (−log (*p* value) >14.2) and granulocyte adhesion and diapedesis (−log (*p* value) >13.1) are the top three canonical pathways dysregulated at all stages following injury (Table 1).

**Figure 2:**
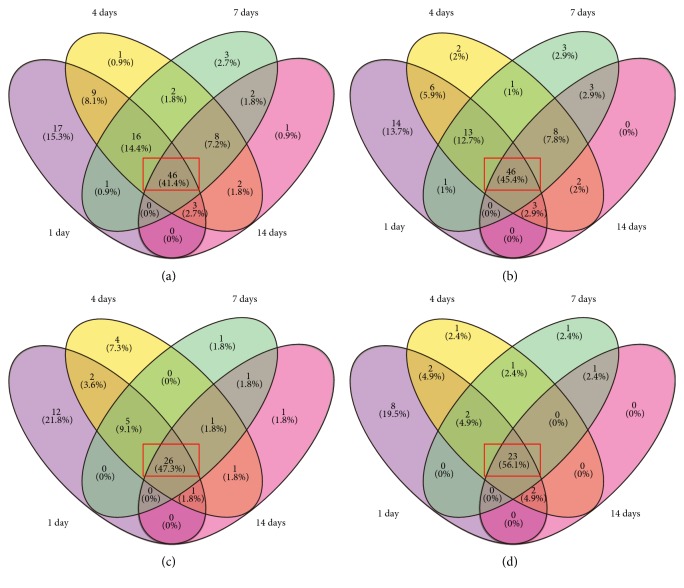
Differential gene expression patterns in the pathways of agranulocyte adhesion and diapedesis (a), granulocyte adhesion and diapedesis (b), IL-6 signaling (c), and IL-10 signaling (d) at 1, 4, 7, and 14 days after injury shown as Venn diagrams. The intersection shows the overlapped DEGs.

When analyzing the DEGs at different time points, we found 46, 46, 26, and 24 genes commonly upregulated or downregulated in the pathways of agranulocyte adhesion and diapedesis, granulocyte adhesion and diapedesis, IL-10 signaling, and IL-6 signaling, respectively (see the intersect in red, Figures 2(a), 2(b), 2(c), and 2(d)). Moreover, we also found that most DEGs in these pathways continuously either upregulated or downregulated, though most genes are upregulated following injury (Additional Files 13, 14, 15, and 16), indicating that all time points post peripheral nerve injury share similar immune/inflammatory reactions.

### 3.3. qRT-PCR Validation of Genes in Immune/Inflammatory-Related Pathways

**Figure 3:**
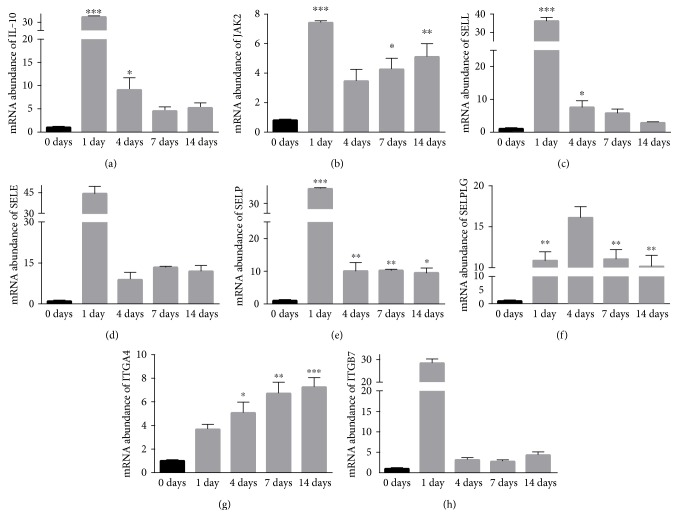
Validation of RNA-seq results by quantitative real-time PCR (qRT-PCR). qRT-PCR was used to determine the differential gene expression at different time points. Data are analyzed with one-way ANOVA and presented as means ± SEM. ^∗^*p* < 0.05, ^∗∗^*p* < 0.01, and ^∗∗∗^*p* < 0.001.

To validate RNA-seq results for individual genes, we performed RT-PCR with different sets of RNA samples used for transcriptome sequencing. In agranulocytes/granulocytes adhesion and diapedesis, inflammatory signals induce endothelial cells to exocytose P-selectin (SELP) and E-selectin (SELE). SELP and SELE bind to their respective ligands P-selectin glycoprotein ligand (SELPLG) and E-selectin ligand-1 (ESL1), mediating cell contact between agranulocytes or granulocytes and endothelial cells. L-selectin (SELL) is recognized by SELE or other molecules, leading to a rolling movement of the agranulocytes/granulocytes on the endothelial cell surface (Additional File 17A). This further activates integrins, including but not limit to, integrin *α*-4 (ITGA4) and ITGB7 (Additional File 17A) [13–15]. IL-6 signaling is critical in proinflammatory response. IL-6 binds to its receptor IL-6R, known as CD126, leading to activation of receptor-associated kinases, for example, Janus kinase 2 (JAK2) (Additional File 17B) [16]. IL-10 is an anti-inflammatory cytokine, which limits and terminates the inflammatory response, including IL-6 signaling (Additional File 17C) [17]. Pathways and molecules are summarized in Table 2. Based on the significant biological functions, we analyzed mRNA expression of SELL, SELE, SELP, SELPG, and ITGA4 in agranulocytes/granulocytes adhesion and diapedesis pathways; ITGB7 in agranulocytes adhesion and diapedesis pathway, JAK2 in IL-6 pathway; and IL-10 from five different time points. mRNA levels of IL-10, SELL, SELE, and ITGB7 were dramatically increased at 1 day after nerve injury but were slowly declined from day 4 (Figures 3(a), 3(c), 3(d), and 3(h)). JAK2, SELP, and SELPLG are sustained to be upregulated during peripheral nerve degeneration and regeneration (Figures 3(b), 3(e), and 3(f)). The expression of ITGA4 starts to increase until 4 days post injury and is continuously upregulated at 7 and 14 days post injury (Figure 3(g)). Most expression changes of these genes from the qRT-PCR results match the RNA-seq results, though RNA-seq is more sensitive to show the continuous upregulation of most genes tested at all different stages (Additional Files 5, 7, 9, and 11).

## 4. Discussion

We used a high-resolution time course analysis of transcriptional profiling to characterize and validate expression changes of genes linked to immune/inflammation reactions in sciatic nerve injury. We found that following peripheral nerve injury, immune and inflammation reactions are among the top enriched cellular functions (also seen in [12]). Signaling pathway analysis suggested that agranulocyte adhesion and diapedesis, granulocyte adhesion and diapedesis pathway, IL-6 signaling, and IL-10 signaling are the major immune/inflammation pathways upregulated during nerve degeneration and regeneration. Moreover, we found that multiple genes in these pathways are continuously upregulated following nerve injury. Our study suggests that recruitment of inflammatory cells to the injury sites is sustained following nerve injury and a more general role of immune/inflammatory system in peripheral nerve injury.

Both our RNA-seq and RT-PCR data show continuous upregulation of JAK2, which is consistent with the activated IL-6 signaling during nerve injury [9, 12], indicating the interaction between IL-6 and JAK2. mRNA expression is rapidly upregulated after day 1, while slowly declined in the following two weeks, comparable to what have been found before [8, 18]. This indicates that IL-10 may play a role rather than anti-inflammatory cytokine which stops proinflammatory signaling, for example, IL-6. Another possibility is discordant protein and mRNA expression of IL-10 [8]. RT-PCR shows that SELE, SELL, ITGA4, ITGB7, and IL-10 are upregulated only at certain time points, but our RNA-seq data reveal that all genes tested are continuously upregulated following injury (Additional Files 5, 7, 9, and 11), indicating RNA-seq is more sensitive to show the statistical difference. Protein levels should be further determined by Western blot. In addition, though these genes are sustained upregulated shown by RNA-seq, the fold changes compared to sham group are temporal-specific. The fold changes of these mRNA molecules in immune/inflammatory pathways might account for the dynamic responses in immune/inflammatory system following injury.

The activation of immune response seems beneficial for peripheral nerve regeneration, based on the observation that regeneration was severely attenuated with elimination of macrophages and neutrophils [1, 19]. The influences of inflammation on the spinal cord, however, are considered as destructive. Some molecules (e.g., TNF) are upregulated and toxic to neurons [20]. In our study, we also find sustained activation of TNF in the injured peripheral nerve (Additional File 5), indicating the single molecule change cannot account for the general role of the immune system in peripheral nerve regeneration. Therefore, our systematic analysis of transcriptome is compelling to study the effects of the immune system in peripheral nerve regeneration.

We find even at 14 days post injury, immune/inflammatory pathways are still active. Whether this activation has negative effects that impede functional recovery requires further analysis. Moreover, further studies should also clarify the complex interactions between these inflammatory mediators and the specific contribution of each to peripheral nerve damage and repair.

The data sets supporting the results of this article are included within the article and its additional files.

## Supplementary Material

Additional file 1. List of primer pairs for qRT-PCR; Additional file 2. Diseases and bio functions of DEGs following nerve injury by IPA analysis; Additional file 3. DEGs in immune cell trafficking following injury; Additional file 4. DEGs in inflammatory response following injury; Additional file 5. DEGs in agranulocyte adhesion and diapedesis following injury; Additional file 6. Schematic network of agranulocyte adhesion and diapedesis signaling following injury; Additional file 7. DEGs in granulocyte adhesion and diapedesis following injury; Additional file 8. Schematic network of granulocyte adhesion and diapedesis signaling following injury; Additional file 9. DEGs in IL-6 signaling following injury; Additional file 10. Schematic network of Il-6 signaling following injury; Additional file 11. DEGs in IL-10 signaling following injury; Additional file 12. Schematic network of Il-10 signaling following injury; Additional file 13. Sciatic injury induced DEGs in agranulocyte adhesion and diapedesis; Additional file 14. Sciatic injury induced DEGs in granulocyte adhesion and diapedesis; Additional file 15. Sciatic injury induced DEGs in IL-6 signaling pathway; Additional file 16. Sciatic injury induced DEGs in IL-10 signaling pathway; Additional file 17. Schematic network of agranulocyte/granulocyte adhesion and diapedesis, IL-6 signaling, and IL-10 signaling.

## Figures and Tables

**Table 1:** Significance of agranulocyte adhesion and diapedesis, granulocyte adhesion and diapedesis, IL-10 signaling, and IL-6 signaling at each time point following peripheral nerve injury. The −log (*p* value) and ratio of these signaling pathways were listed.

Agranulocyte adhesion and diapedesis	Rank	−log (*p* value)	Ratio	Number of differentially expressed genes

1 day	1	16.5	0.487	92
4 days	1	14.2	0.460	87
7 days	2	17.7	0.413	78
14 days	3	14.6	0.328	62

Granulocyte adhesion and diapedesis	Rank	−log (*p* value)	Ratio	Number of differentially expressed genes

1 day	2	13.7	0.469	83
4 days	3	13.1	0.458	81
7 days	1	17.9	0.424	75
14 days	1	16.1	0.350	62

IL-6 signaling	Rank	−log (*p* value)	Ratio	Number of differentially expressed genes

1 day	29	5.35	0.397	46
4 days	91	3.36	0.345	40
7 days	53	4.20	0.293	34
14 days	48	5.44	0.267	31

IL-10 signaling	Rank	−log (*p* value)	Ratio	Number of differentially expressed genes

1 day	7	8.74	0.544	37
4 days	38	5.40	0.456	31
7 days	22	6.78	0.412	28
14 days	30	8.07	0.382	26

**Table 2:** Molecules verified and pathways involved.

Molecules verified	Pathways	Function
SELP, SELE, SELPLG, SELL, ITGA4	Agranulocyte/granulocyte adhesion and diapedesis	Extravasation of agranulocyte/granulocyte, including (1) tethering, (2) rolling and activation, (3) firm adhesion to the endothelium, (4) diapedesis, and (5) transendothelial migration
ITGB7	Agranulocyte adhesion and diapedesis	
JAK2	IL-6	Proinflammatory response
IL-10	IL-10	Anti-inflammatory response
