# Supplementary figures and images for: Transcriptional Profiling at High Temporal Resolution Reveals Robust Immune/Inflammatory Responses during Rat Sciatic Nerve Recovery
Source: Mediators Inflamm. 2017 Apr 12;2017:3827841. doi: 10.1155/2017/3827841 (PMC5405595; doi:10.1155/2017/3827841)

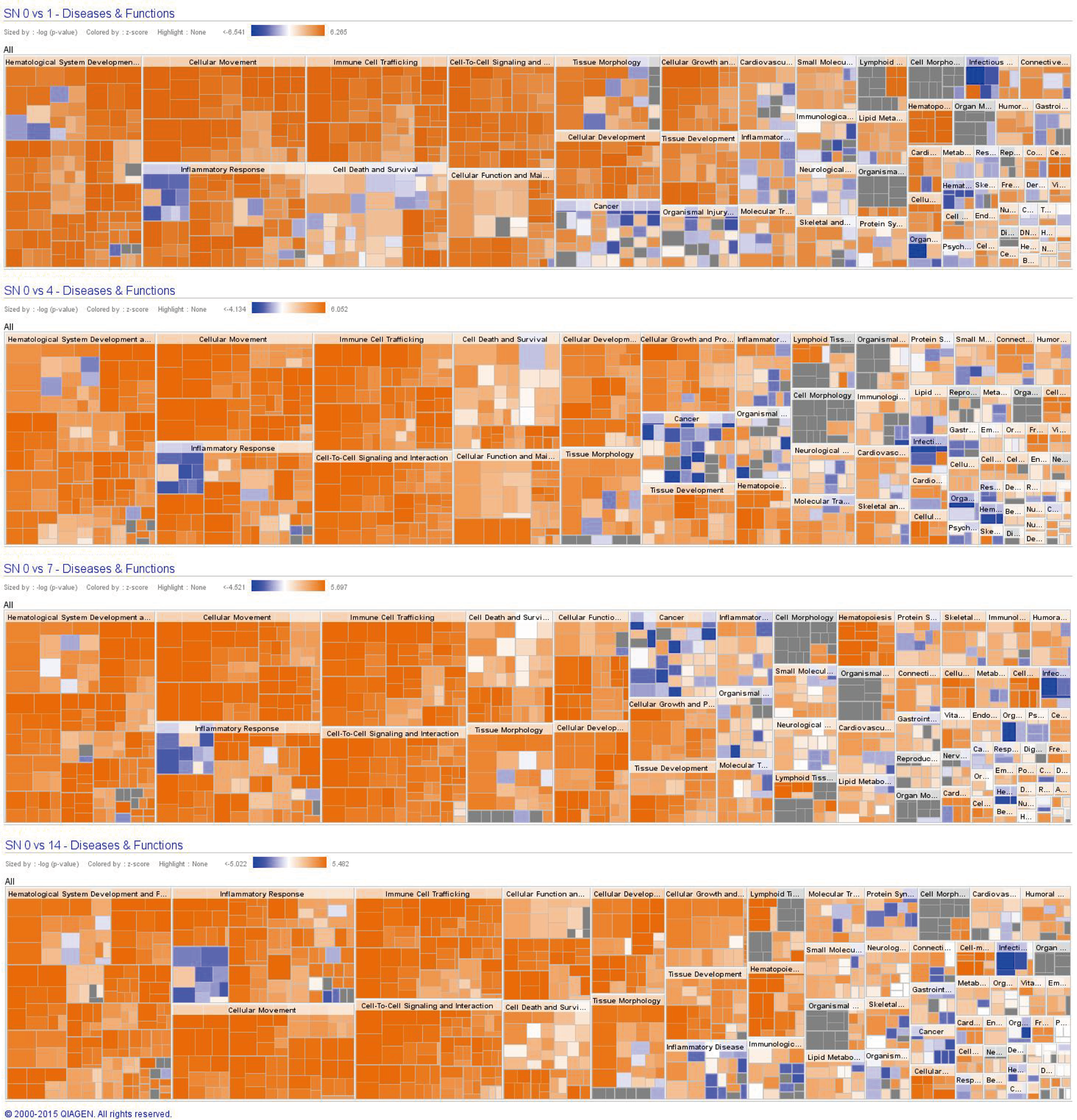

Supplement: Supplementary file 2 [file 3827841.f2.tif]

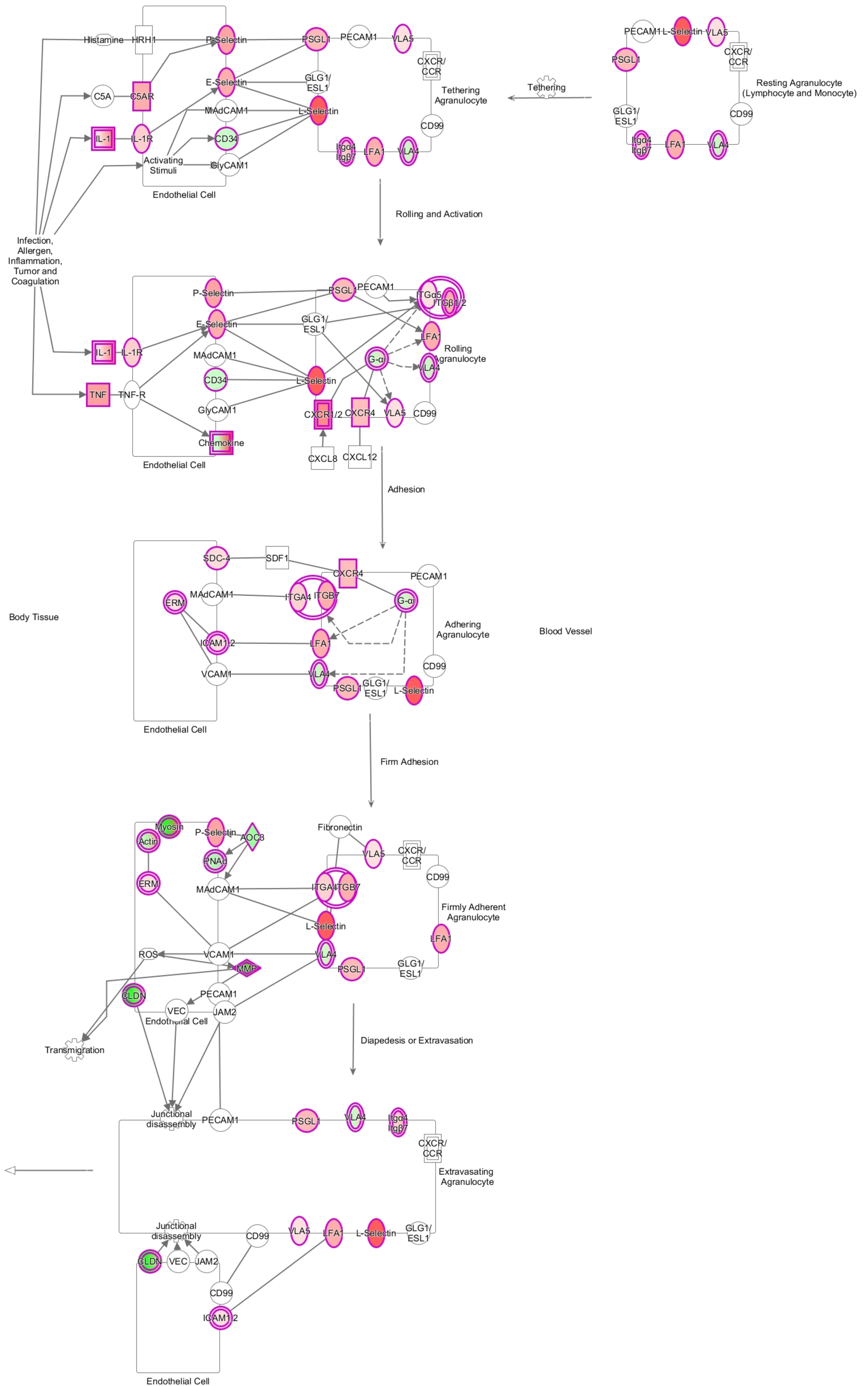

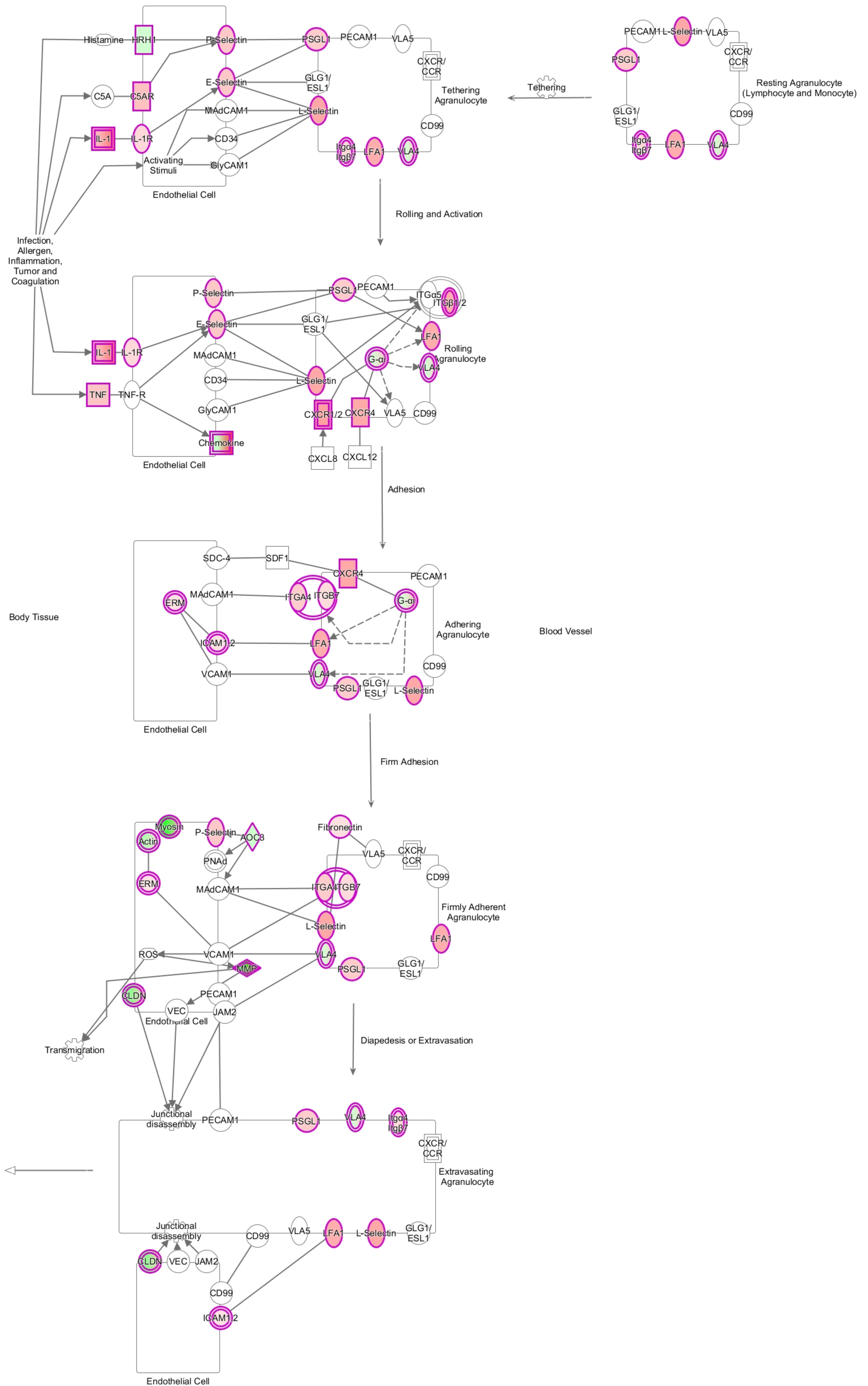

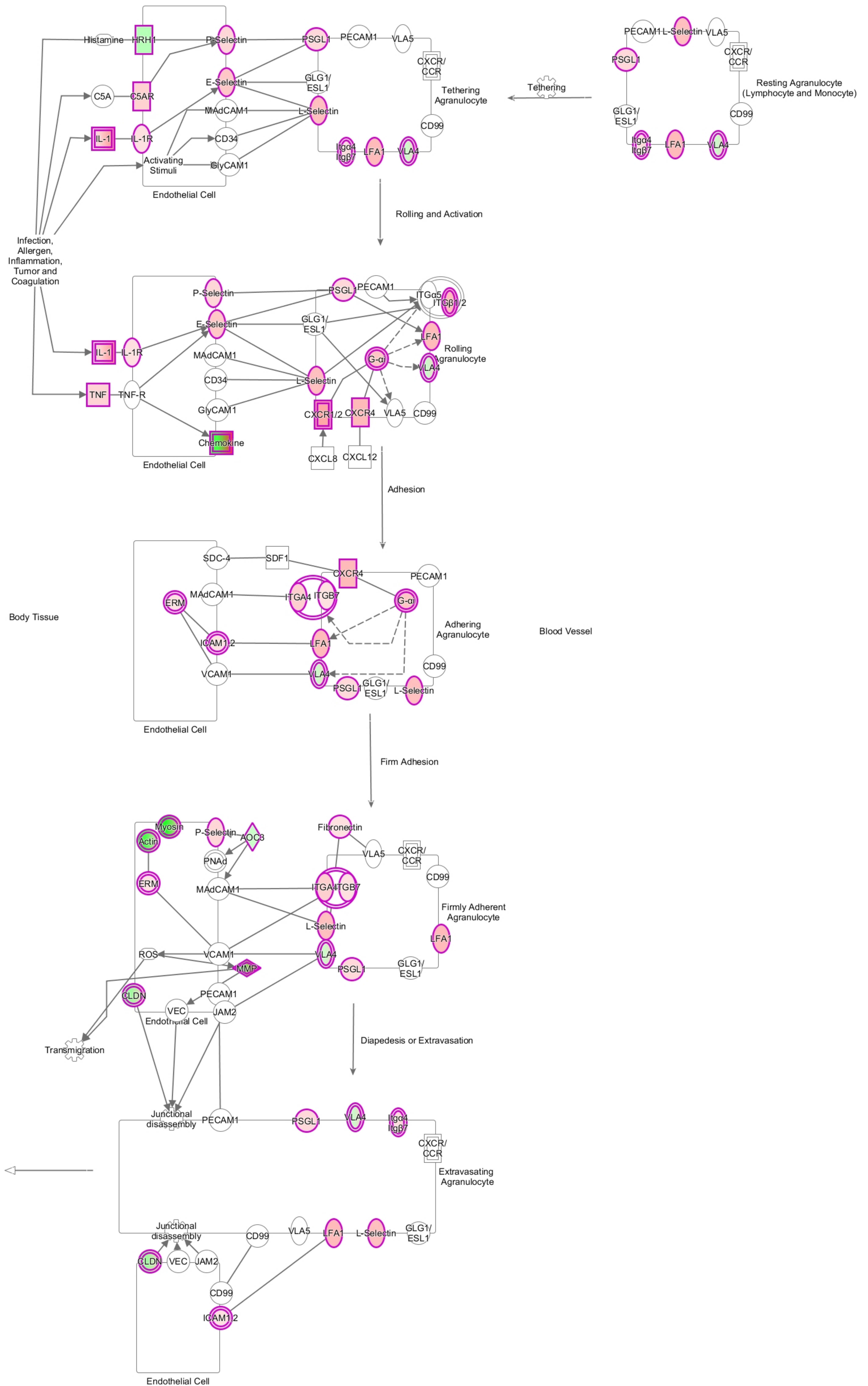

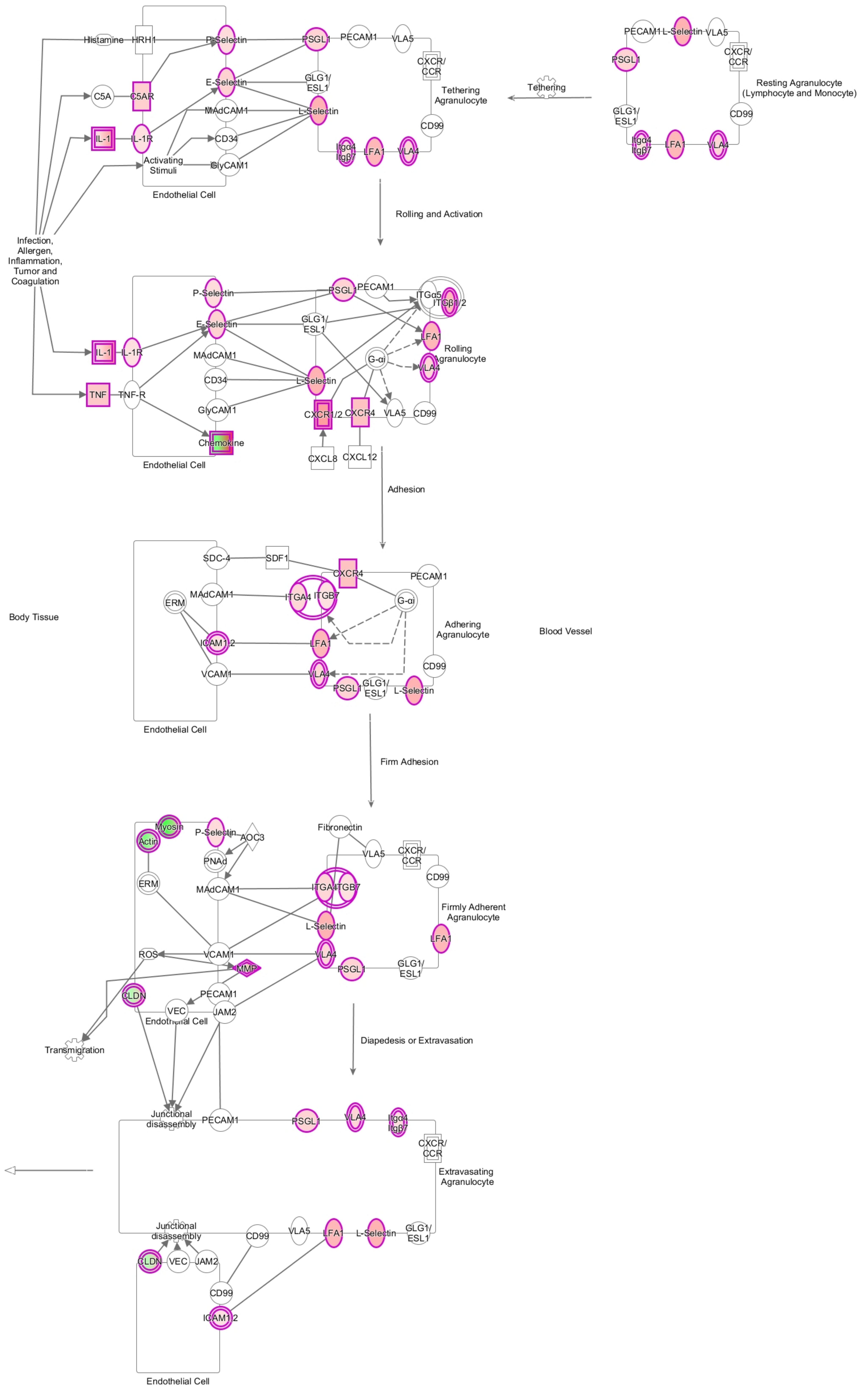

Supplement: Supplementary file 6 [file 3827841.f6.pdf]

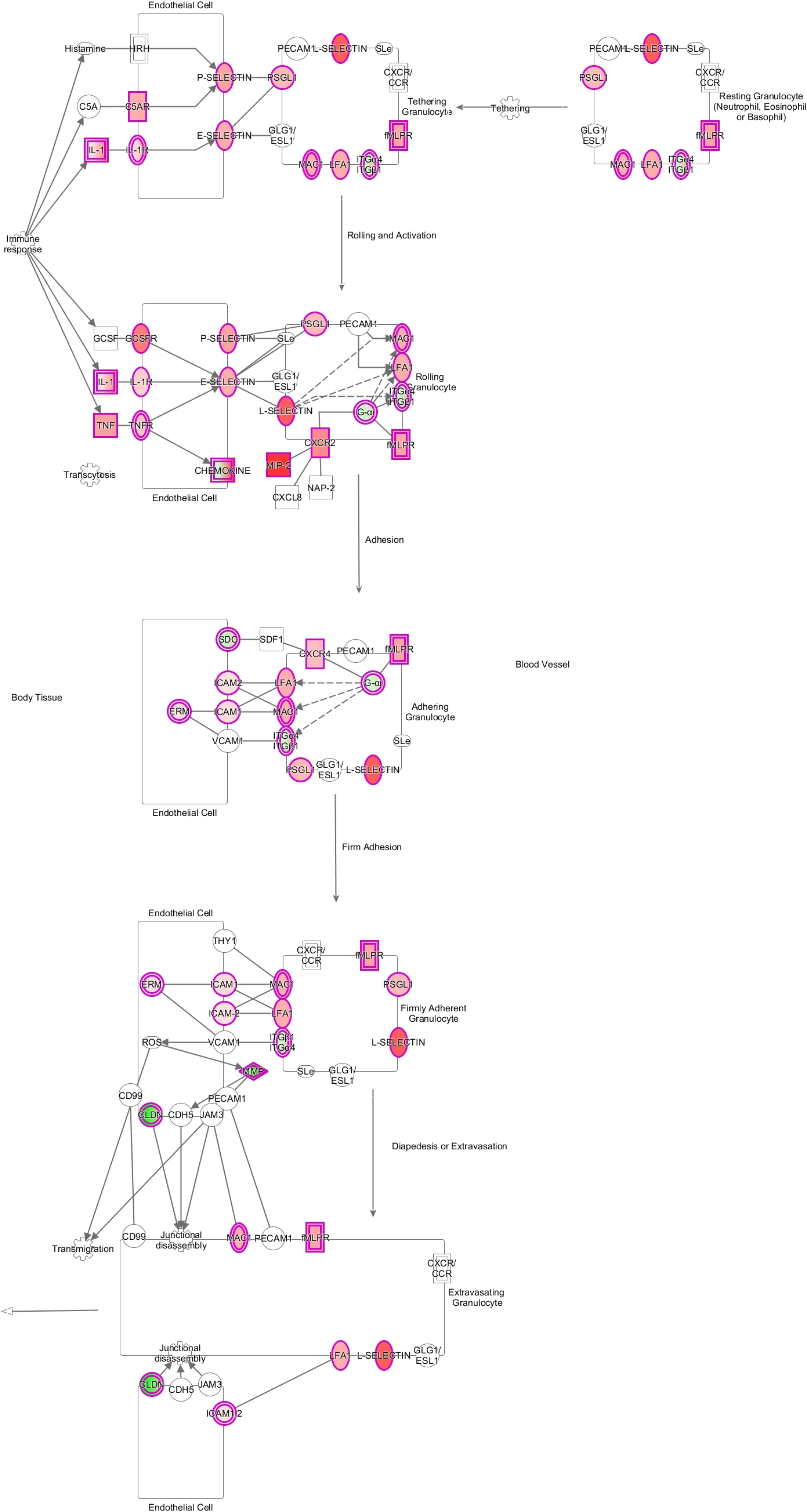

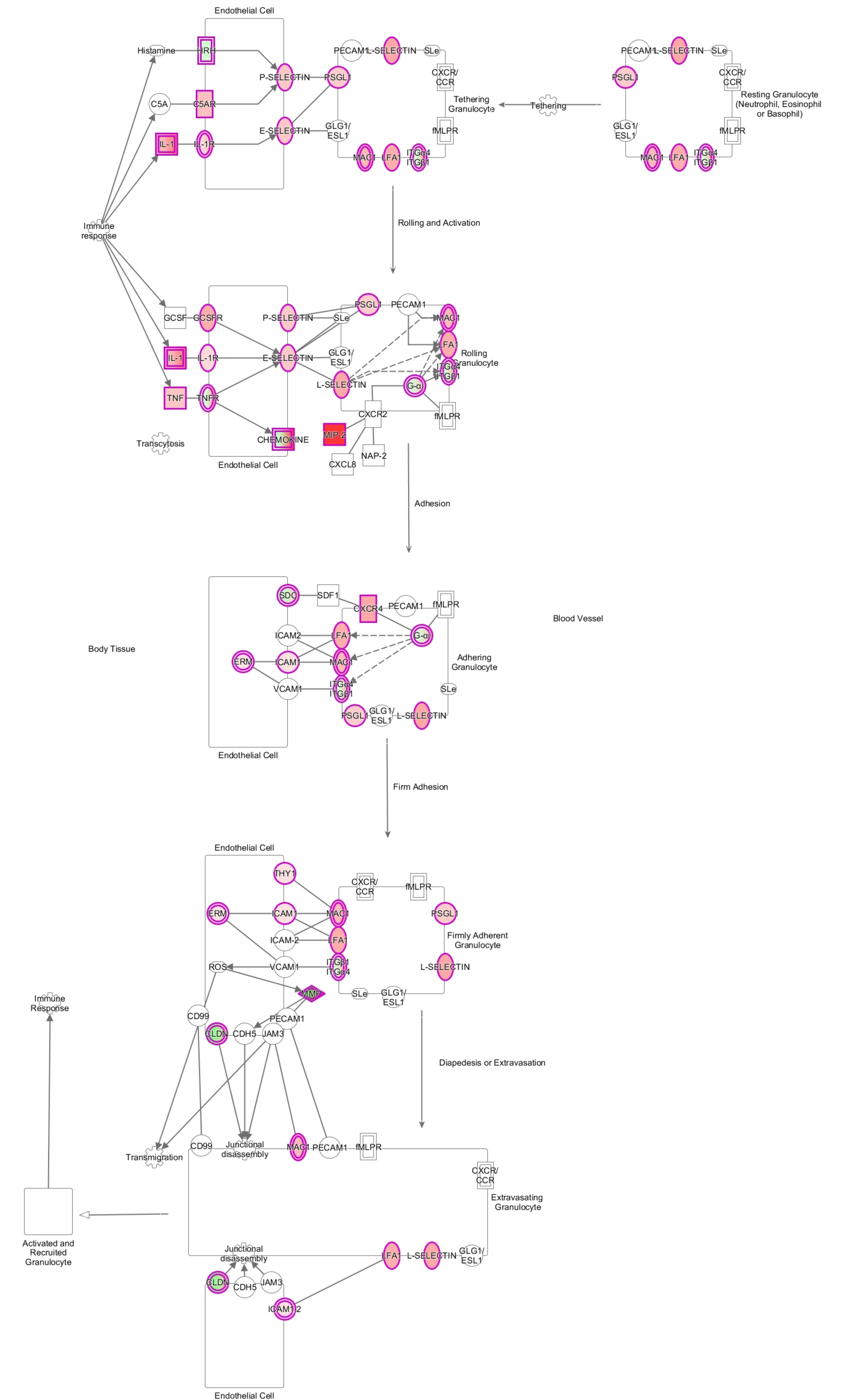

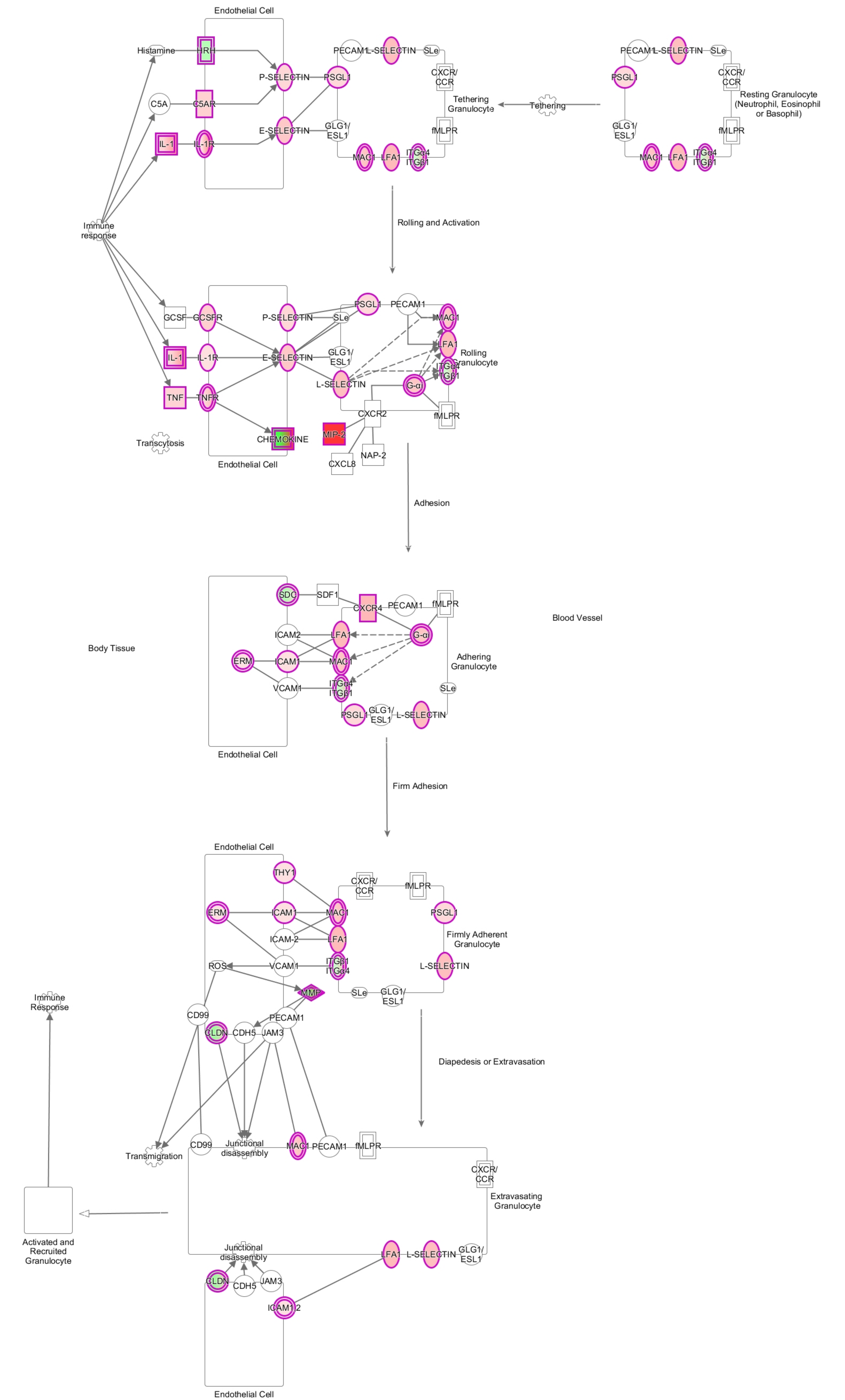

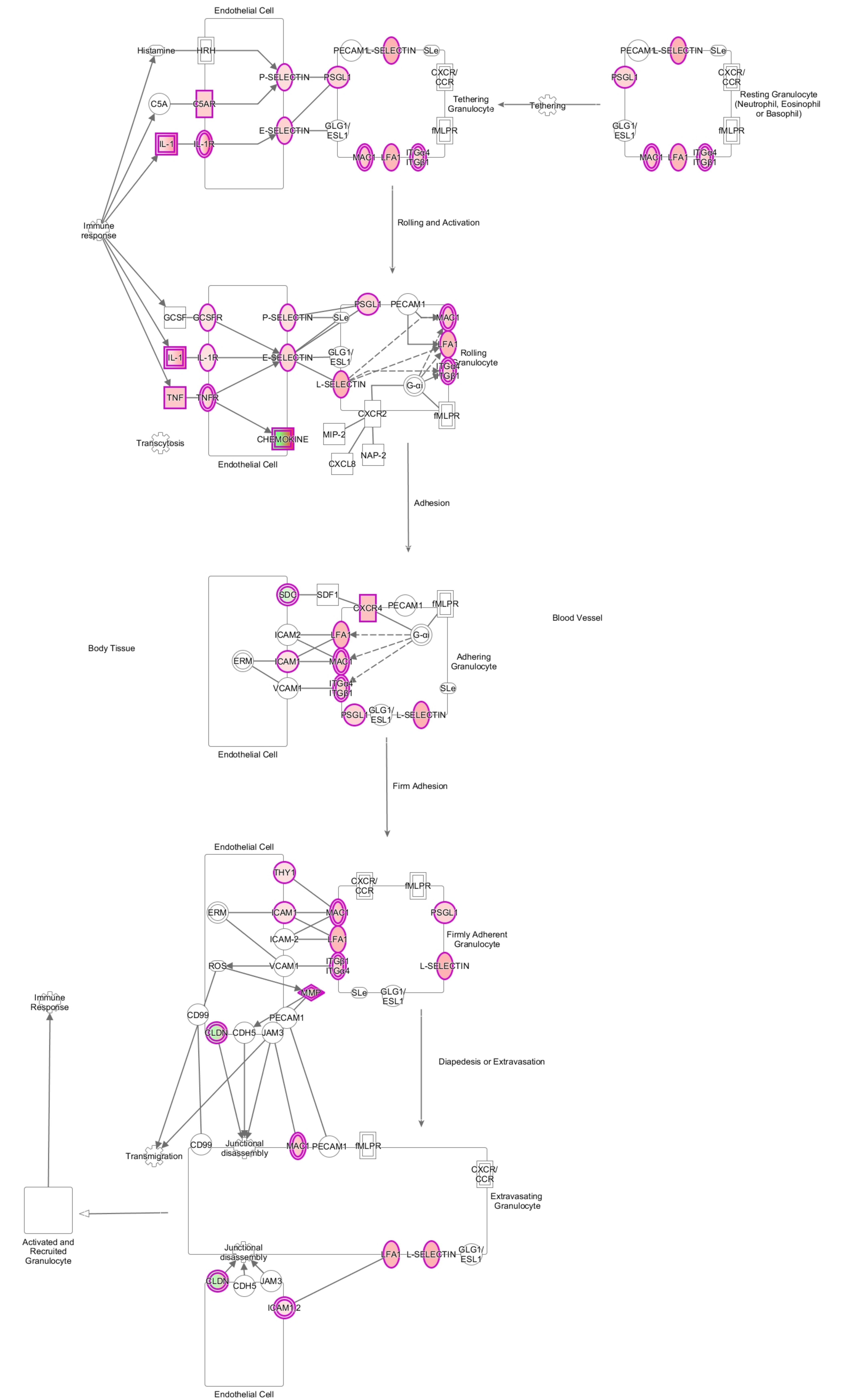

Supplement: Supplementary file 8 [file 3827841.f8.pdf]

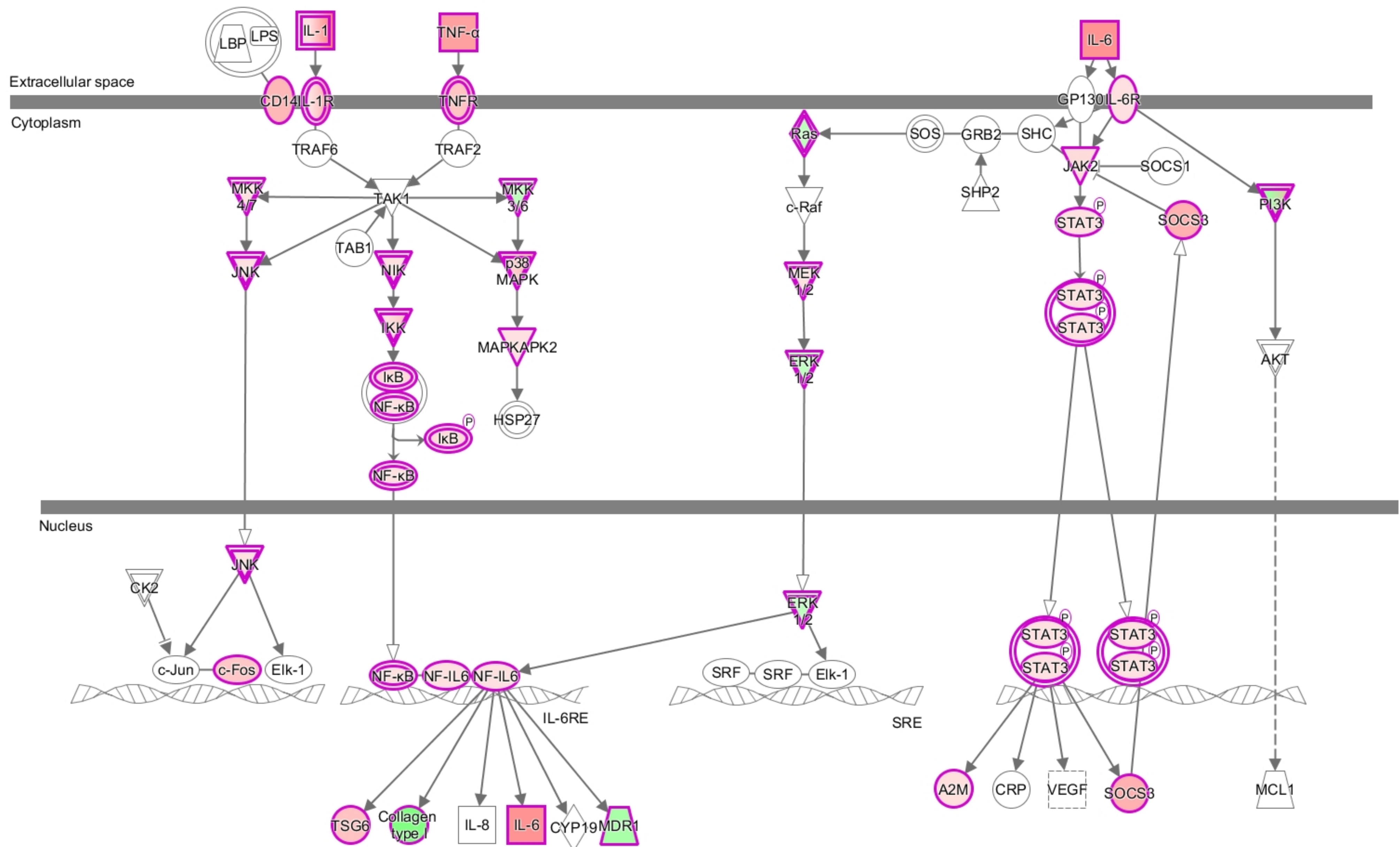

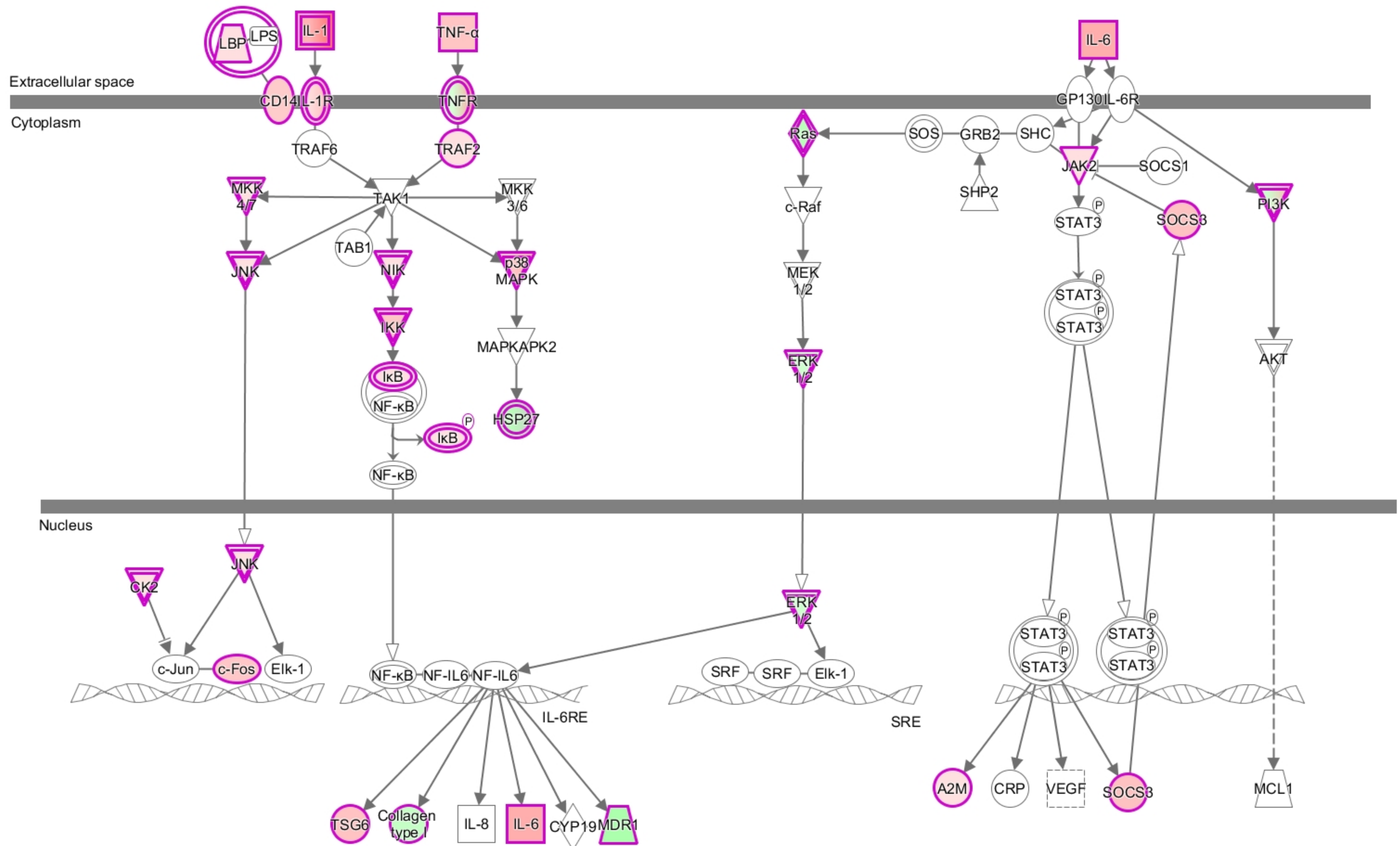

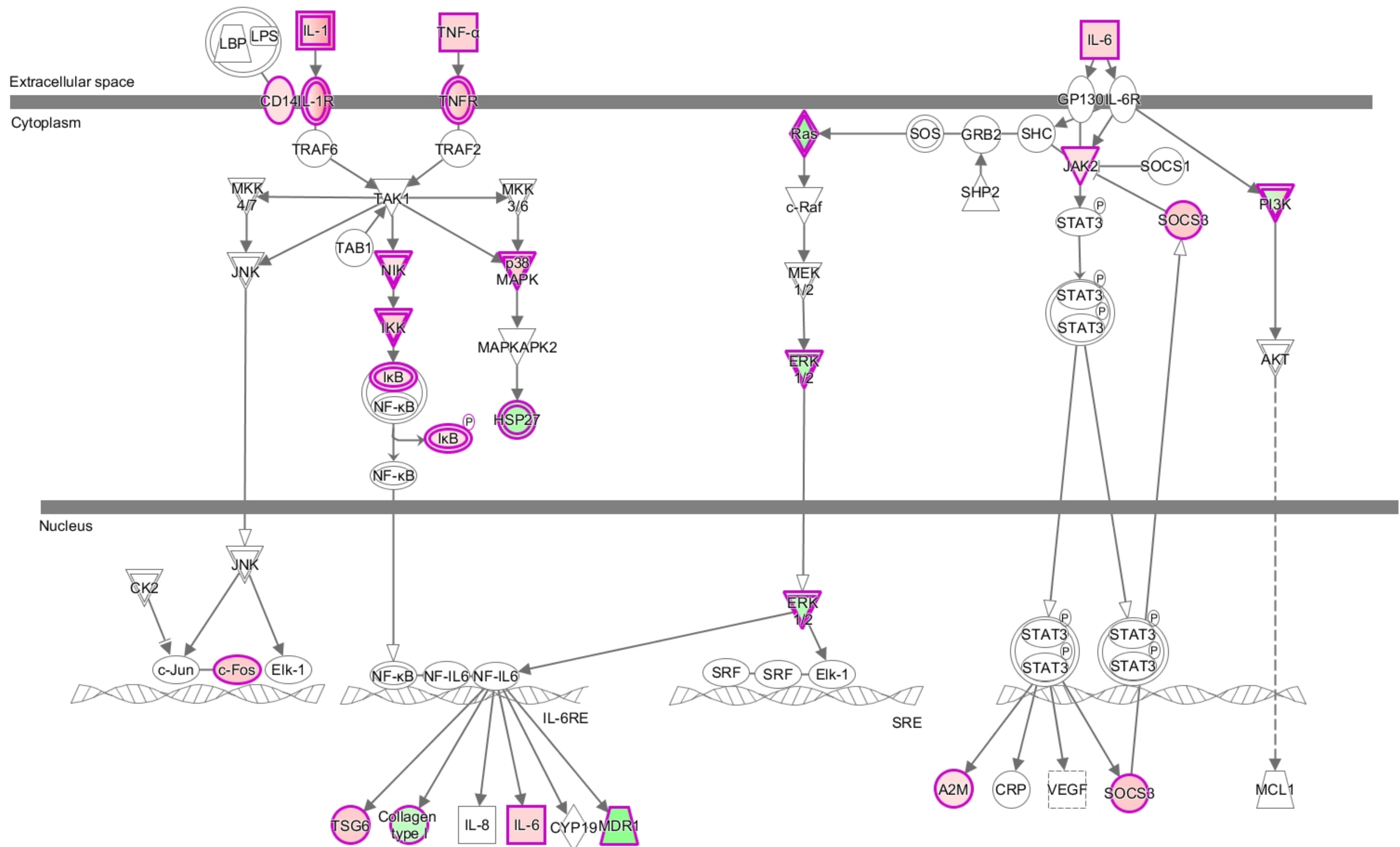

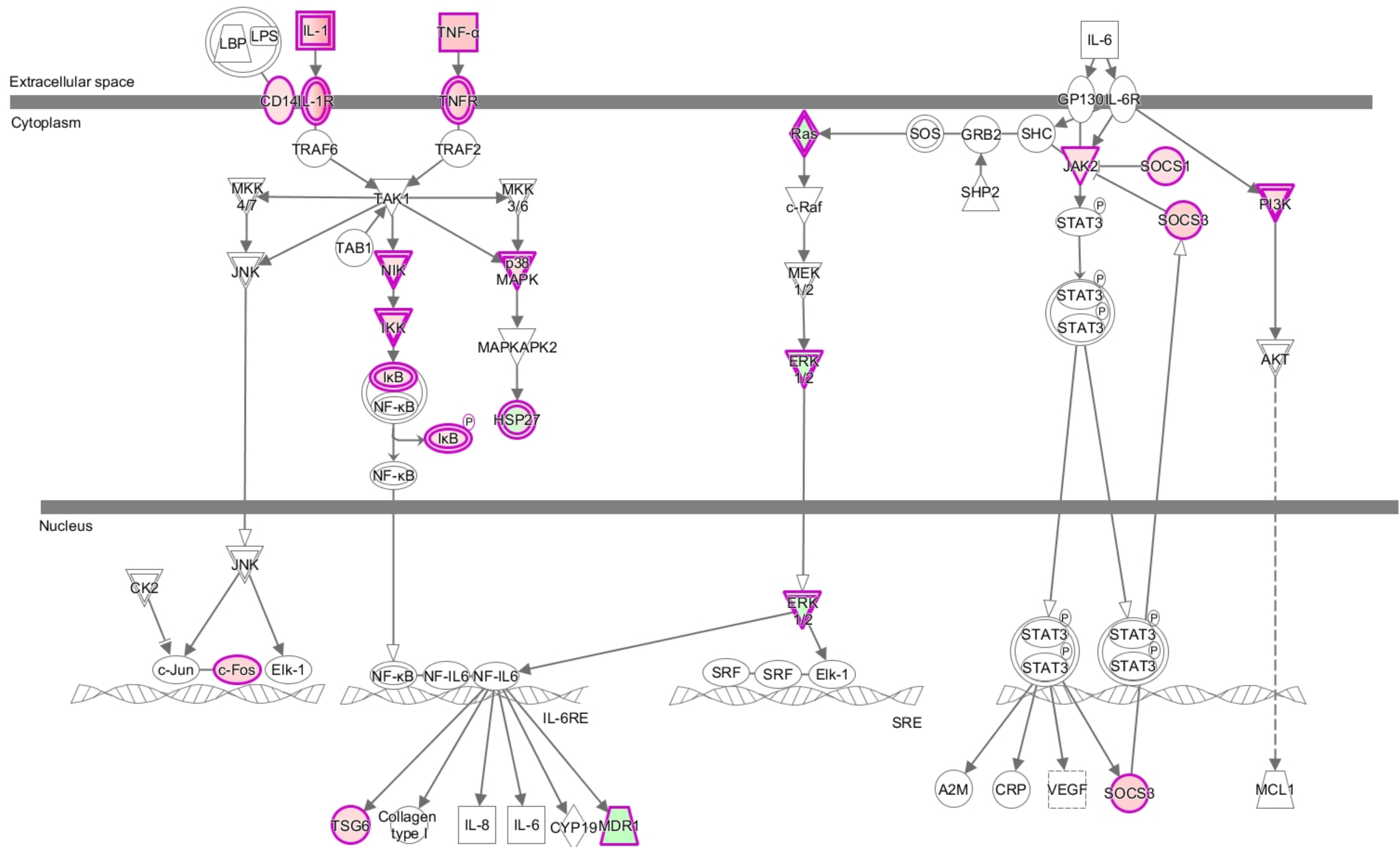

Supplement: Supplementary file 10 [file 3827841.f10.pdf]

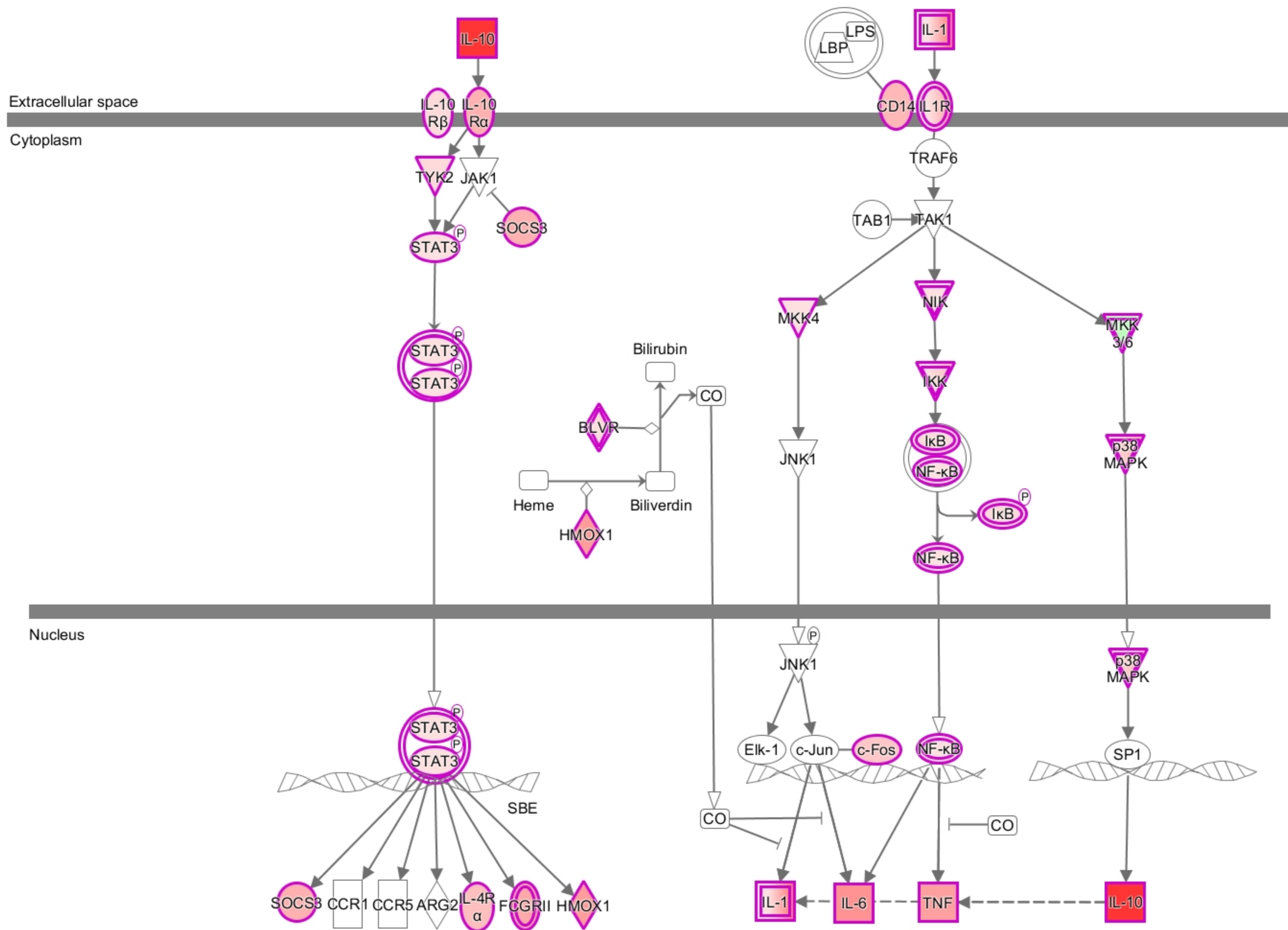

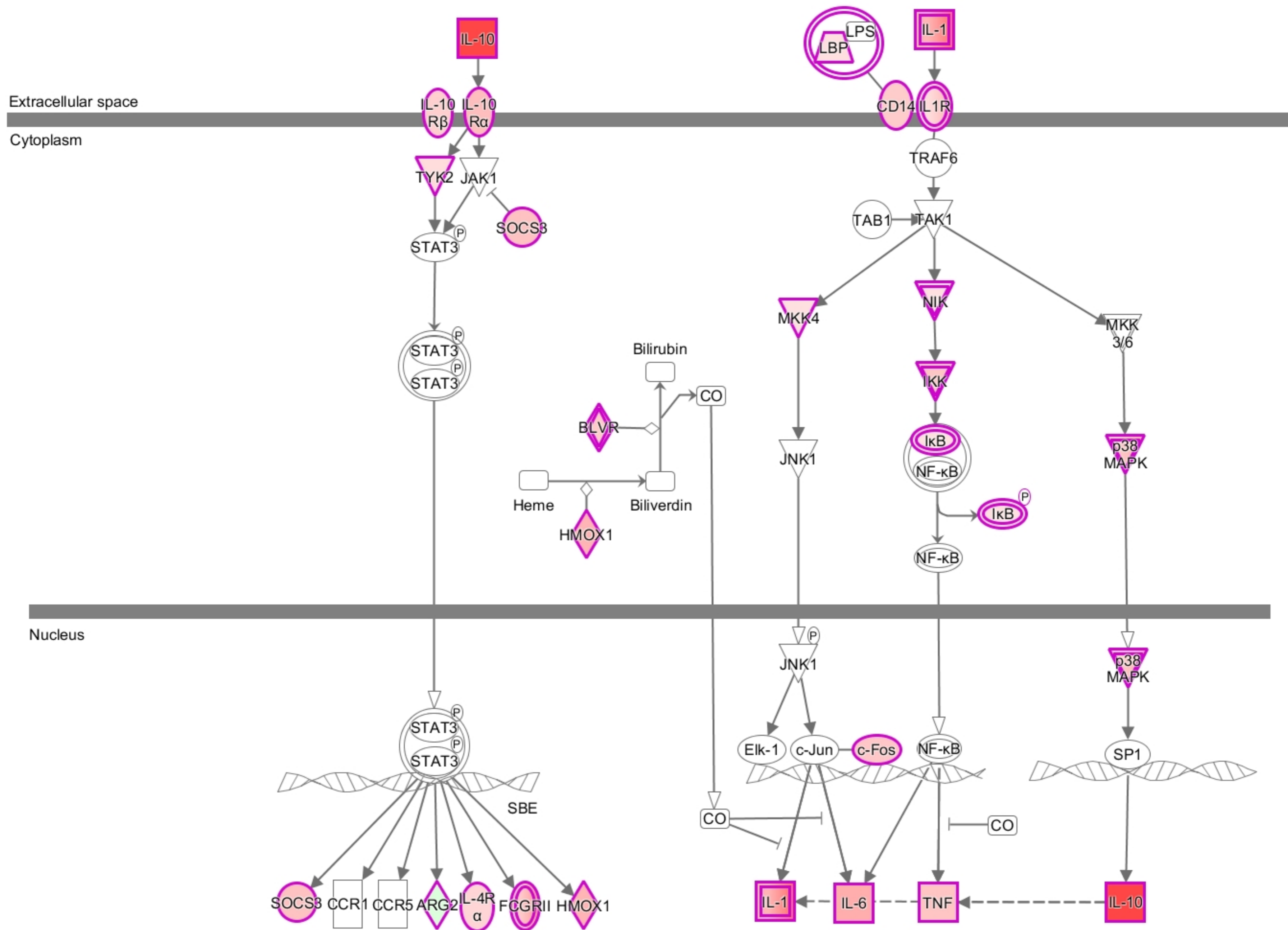

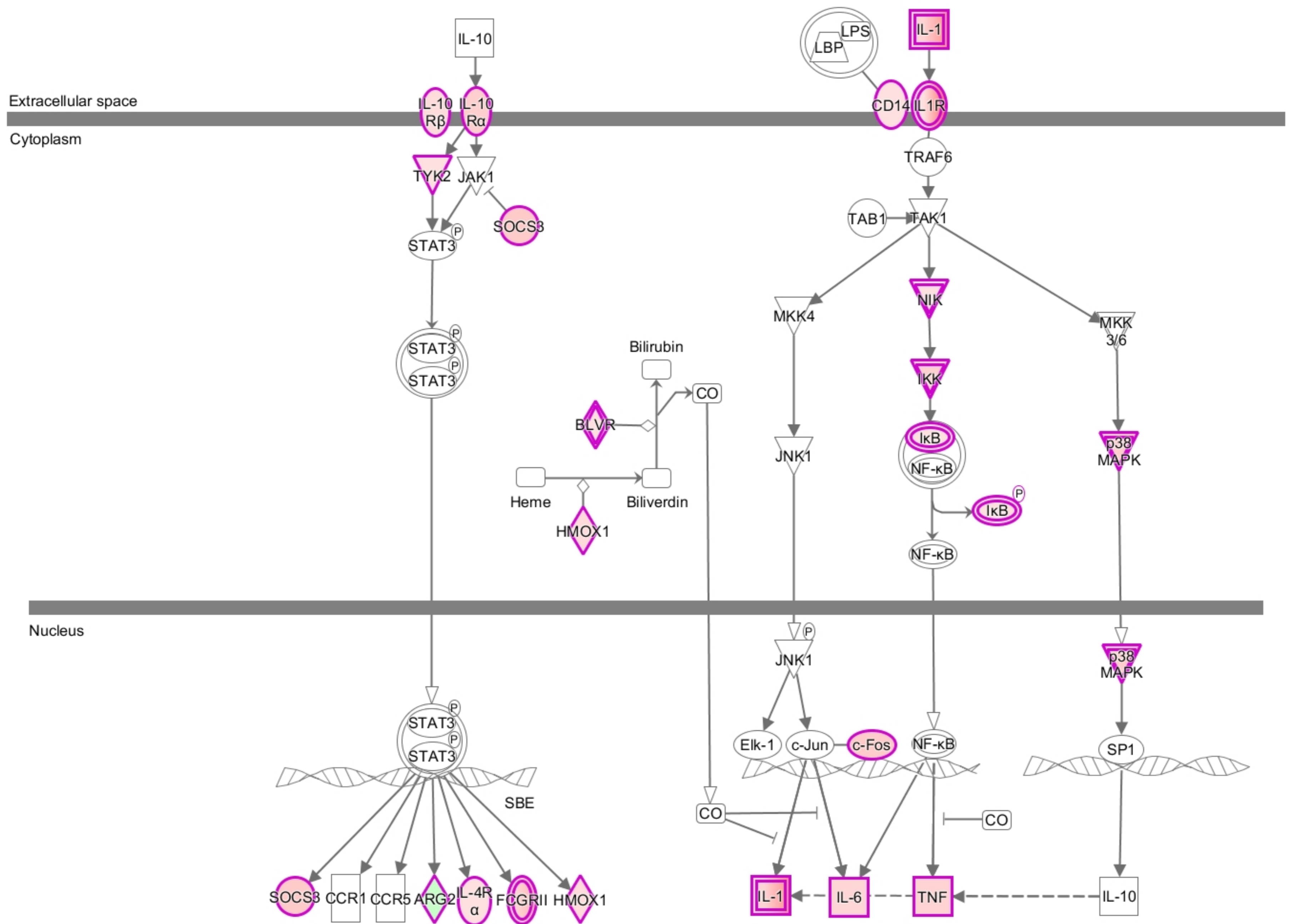

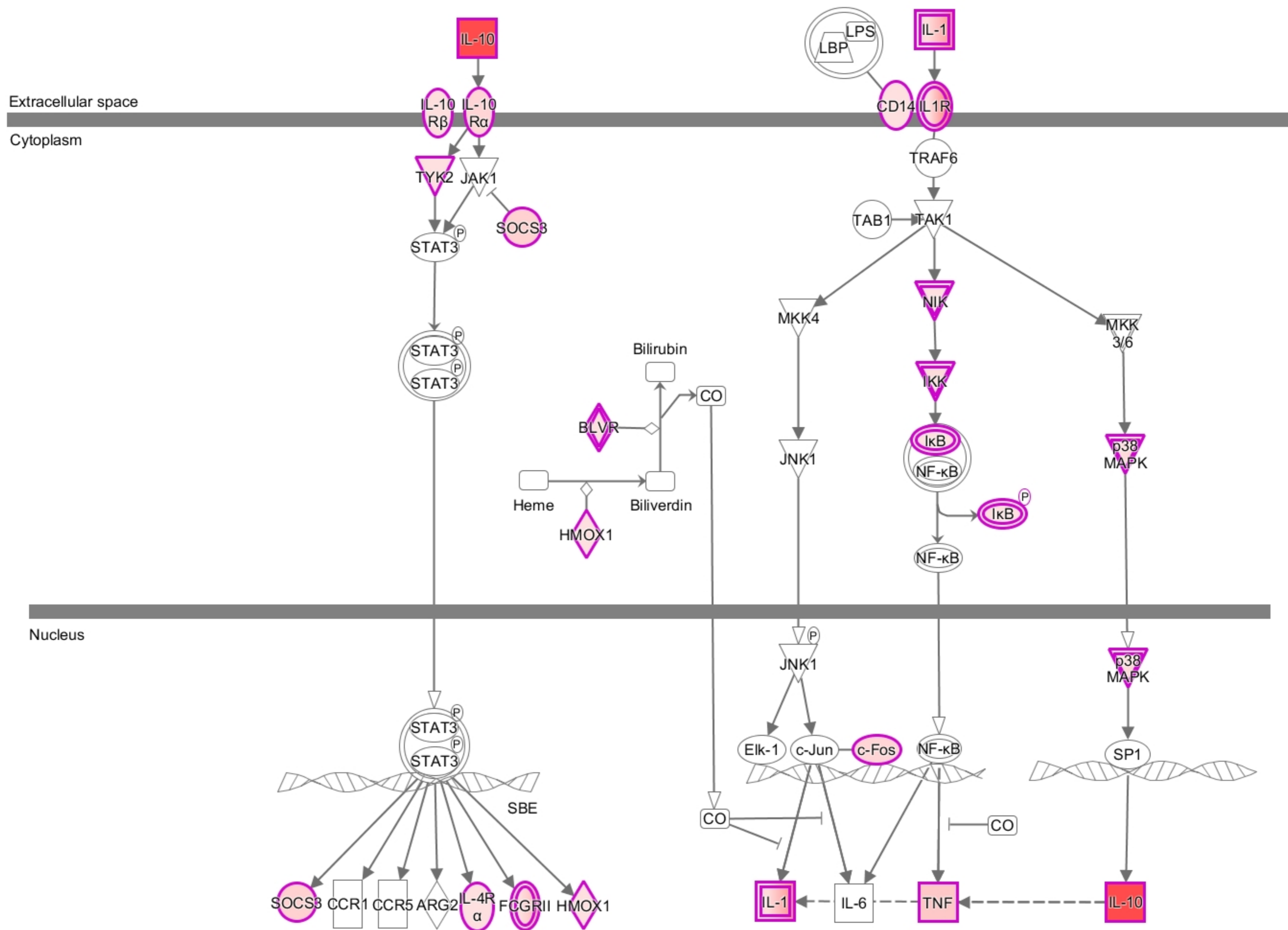

Supplement: Supplementary file 12 [file 3827841.f12.pdf]

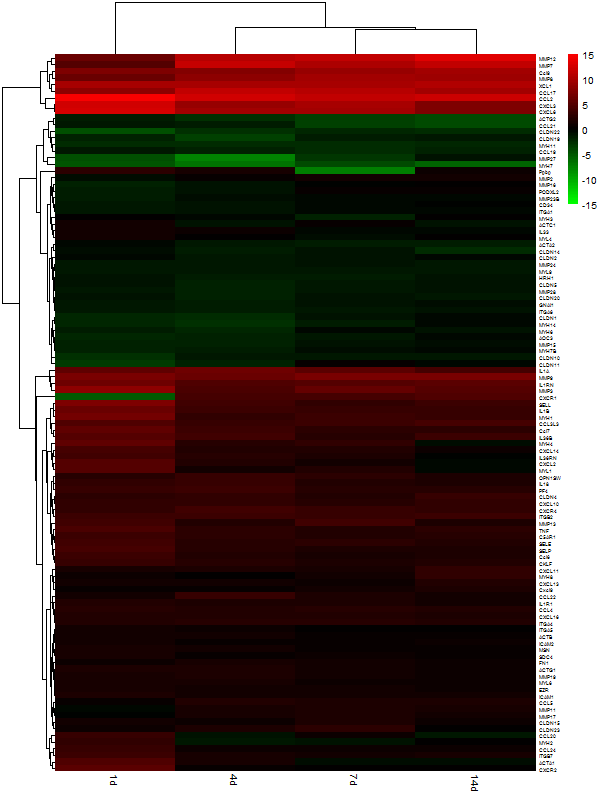

Supplement: Supplementary file 13 [file 3827841.f13.tiff]

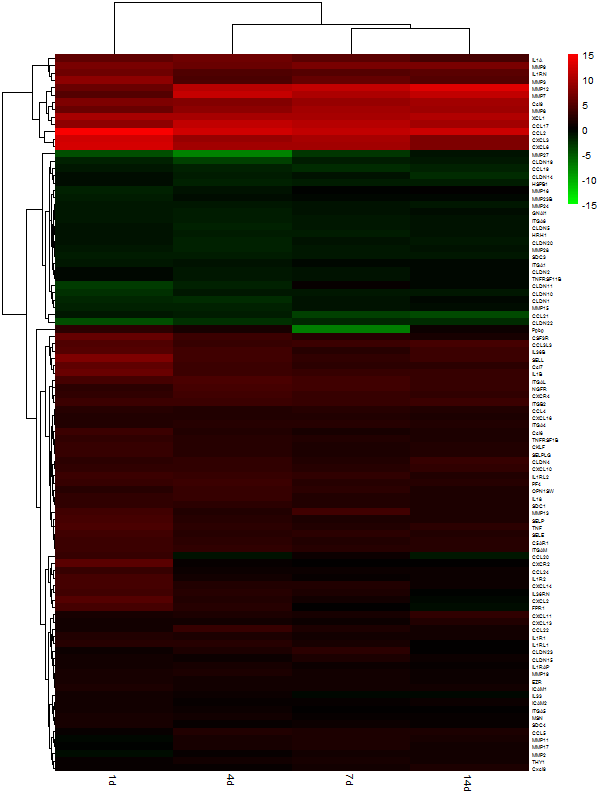

Supplement: Supplementary file 14 [file 3827841.f14.tiff]

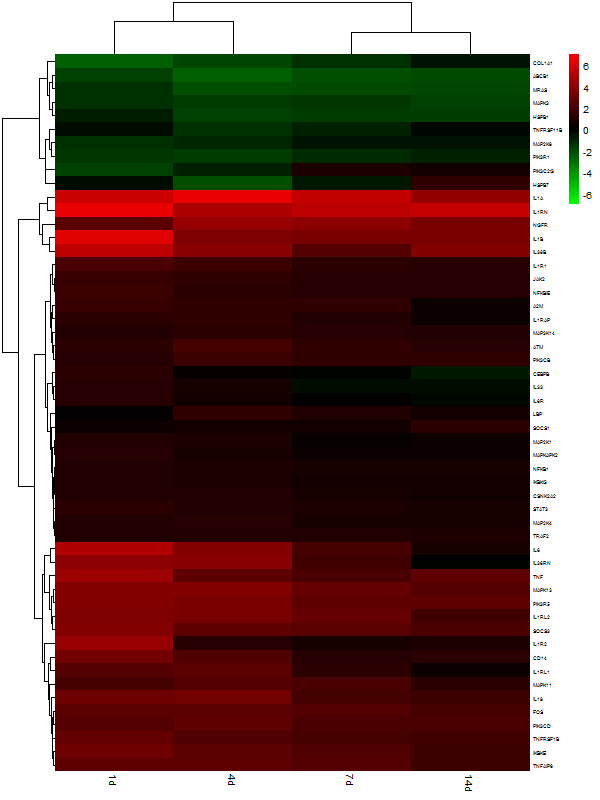

Supplement: Supplementary file 15 [file 3827841.f15.tiff]

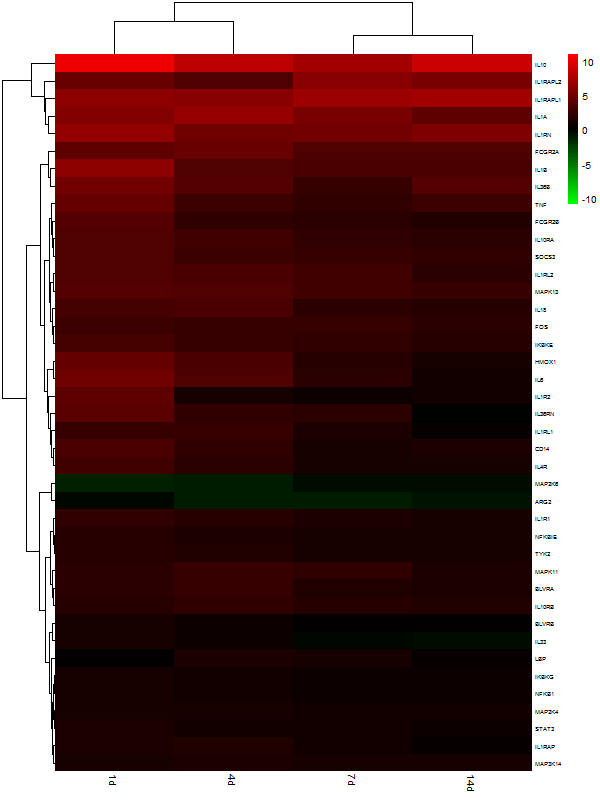

Supplement: Supplementary file 16 [file 3827841.f16.tiff]

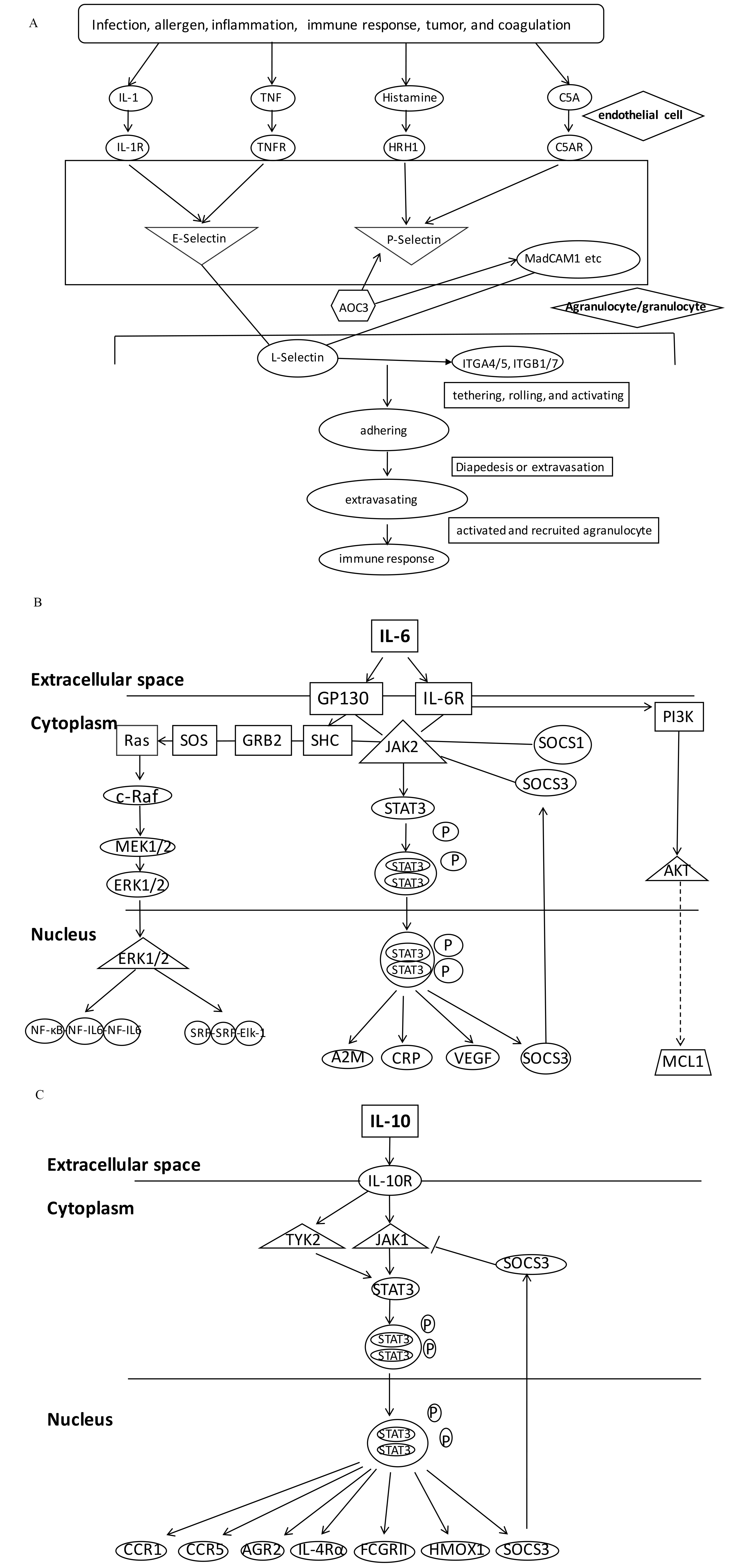

Supplement: Supplementary file 17 [file 3827841.f17.tif]
